# In vivo cardiac diffusion tensor imaging in free-breathing conditions

**DOI:** 10.1186/1532-429X-15-S1-P231

**Published:** 2013-01-30

**Authors:** Hongjiang Wei, Magalie Viallon, Benedicte M Delattre, Vinay M Pai, Han Wen, Hui Xue, Christoph Guetter, Marie-Pierre Jolly, Pierre Croisille, Yuemin Zhu

**Affiliations:** 1 Creatis; CNRS (UMR 5220), Inserm (U1044);, Insa Lyon, Villeurbanne, France; 2 Department of Radiology, University Hospitals of Geneva, Geneva, Switzerland; 3 Imaging Physics Lab, BBC/NHLBI/NIH, Bethesda, MD, USA; 4 Siemens Corporate Research, Princeton, NJ, USA; 5 Jean-Monnet University, Saint-Etienne, France

## Background

The fiber structure of the human heart contributes significantly to efficient ventricular function in the presence of disease. DTI provides a non-invasive approach for the three-dimensional depiction of the myocardial fiber architecture. The biggest problem for in vivo cardiac DTI is the signal loss caused by motion. Recently, to cope with human physiological motion problem, a robust method called PCATMIP was proposed (Rapacchi, Invest radiol 2011) that uses principal component analysis (PCA) filtering to improve the signal-to-noise ratio (SNR) and temporal maximum intensity projection (TMIP) approach to compensate the signal loss. While performing cardiac DTI during subject's breath-hold may be not realistic to apply in clinical routine, achieving acquisitions during subject free-breathing represents an ultimate objective. In this study, our objective was to obtain in vivo DTI parameters of the human heart with free-breathing.

## Methods

To cope with intensity fluctuations arising due to motion, our strategy was to acquire multiple diffusion weighted (DW) images at different time points during the diastole in each consecutive cardiac cycle; after each time frame was acquired, the trigger delay was increased by 10ms. At each trigger delay, we obtained 12 direction DW images and b=0. We acquired 10 DT-MRI slices across the whole heart. The total scan time is about 20 minutes at an average heart rate of 60bpm. The MRI parameters are: TE/TR=51/100ms, spatial resolution=2.6x2.6x6mm3, acceleration rate=2 (GRAPPA), partial Fourier=6/8, base resolution matrix=90x160, and b=200s/mm2. Free-breathing DWI scans were then registered using a non-rigid registration algorithm that preserves high accuracy and consistency of the data. Finally, PCATMIP algorithm was applied to the registered images to obtain motion-reduced ones.

## Results

**Figure 1:**
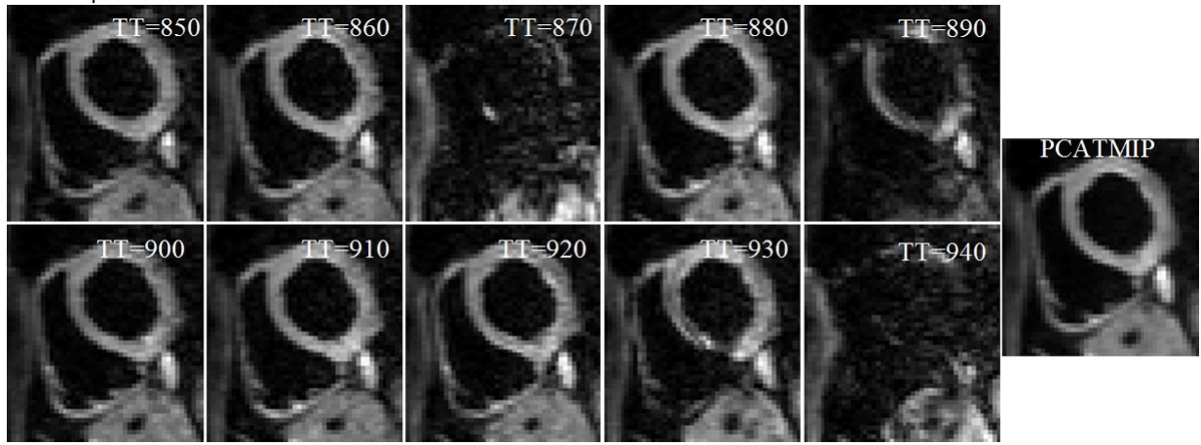
Free-breathing cardiac DW images from 10 repetitions acquired at different time points and processed using PCATMIP.

**Figure 2:**
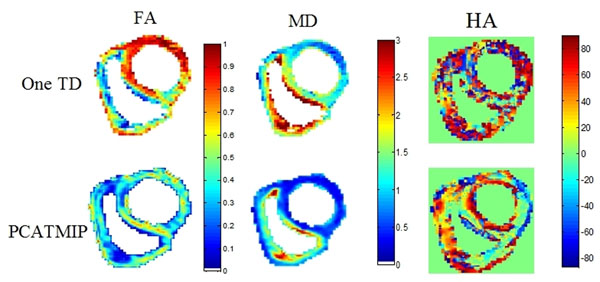
FA, MD, and HA maps comparison between one trigger delay and PCATMIP, the chosen reference trigger delay was the one that exhibited the highest homogeneity and signal intensity in the myocardium.

Lost signal was recovered and the intensity of the DW images was substantially enhanced (Fig. 1). After processing by PCATMIP, both FA (0.43±0.05) and MD (0.75±0.12×10-3 mm2/s) are smaller than those obtained from 1TD acquisition (0.56±0.05 and 1.37±0.16×10-3 mm2/s, respectively) measured over the LV. Coherent helix angle (HA) variations across the ventricle's wall were observed (Fig. 2). The myocardial fiber orientation of the LV showed a circularly symmetric pattern, which reflects the rotation characteristic of cardiac fibers.

## Conclusions

This study demonstrates the feasibility of in vivo cardiac DTI in healthy volunteers. The PCATMIP can be used to minimize the motion-induced signal loss. The proposed acquisition and processing scheme allow to obtain the in vivo DTI parameters while the subject was freely breathing, which opens interesting perspectives for in vivo cardiac DTI clinical applications.

## Funding

This work was supported by the French ANR 2009 (under ANR-09-BLAN-0372-01).

